# Rapidly reversible multiorgan failure after ingestion of “Molly” (pure 3,4-methylenedioxymethamphetamine): a case report

**DOI:** 10.1186/1752-1947-8-204

**Published:** 2014-06-18

**Authors:** Trupti Vakde, Manuel Diaz, Kalpana Uday, Richard Duncalf

**Affiliations:** 1 Division of Pulmonary and Critical Care Medicine, Bronx Lebanon Hospital Center, Bronx, NY, USA; 2 Department of Internal Medicine, Bronx Lebanon Hospital Center, Bronx, NY, USA; 3 Division of Nephrology, Bronx Lebanon Hospital Center, Bronx, NY, USA

**Keywords:** MDMA, “Molly”, Multiorgan failure

## Abstract

**Introduction:**

“Molly” is a street name for pure 3,4-methylenedioxymethamphetamine, an amphetamine derivative which acts by enhancing the release of neurotransmitters such as serotonin, dopamine and norepinephrine. This produces euphoria, increased sensory awareness and central stimulation that make it a popular club drug. Nevertheless, it is also associated with serious side effects. We report an unusual case of rapid multiorgan failure after ingestion of “Molly”. Unlike previously described patterns of 3,4-methylenedioxymethamphetamine-related organ failure, our case does not appear to be related to hyperthermia, rhabdomyolysis or hyponatremia.

**Case presentation:**

A 24-year-old Hispanic man presented to our hospital with an episode of seizure and subsequently developed acute kidney injury, respiratory failure requiring mechanical ventilation and congestive heart failure after ingestion of “Molly”. He rapidly recovered with supportive care and was discharged home.

**Conclusions:**

The spectrum of complications associated with 3,4-methylenedioxymethamphetamine is wide and patient presentation may vary. Moreover, there appears to be multiple mechanisms involved in organ failure. Drug toxicity should be suspected while evaluating a patient with multisystem organ failure of unclear etiology. Treatment is generally supportive sometimes requiring mechanical ventilation and hemodialysis. Nevertheless, complete reversal of organ failure can be expected.

## Introduction

“Molly” is a new club drug gaining popularity in the last decade in all-night dance events and music festivals. The name “Molly” is derived from the term “molecule” and is supposedly the pure form of 3,4-methylenedioxymethamphetamine (MDMA) which was previously popular as “ecstasy”. It is usually distributed as a capsule containing the crystalline powder form of MDMA; it has stimulant and hallucinogenic properties resulting in mood enhancement and euphoria. “Molly” is perceived to be safer than ecstasy because it lacks adulterants, and like ecstasy has limited addictive potential [1,2]. It is now clear that there is significant morbidity associated with this drug including several reported cases of death. We report a case of rapidly reversible multiorgan failure after ingestion of “Molly”.

## Case presentation

**Figure 1:**
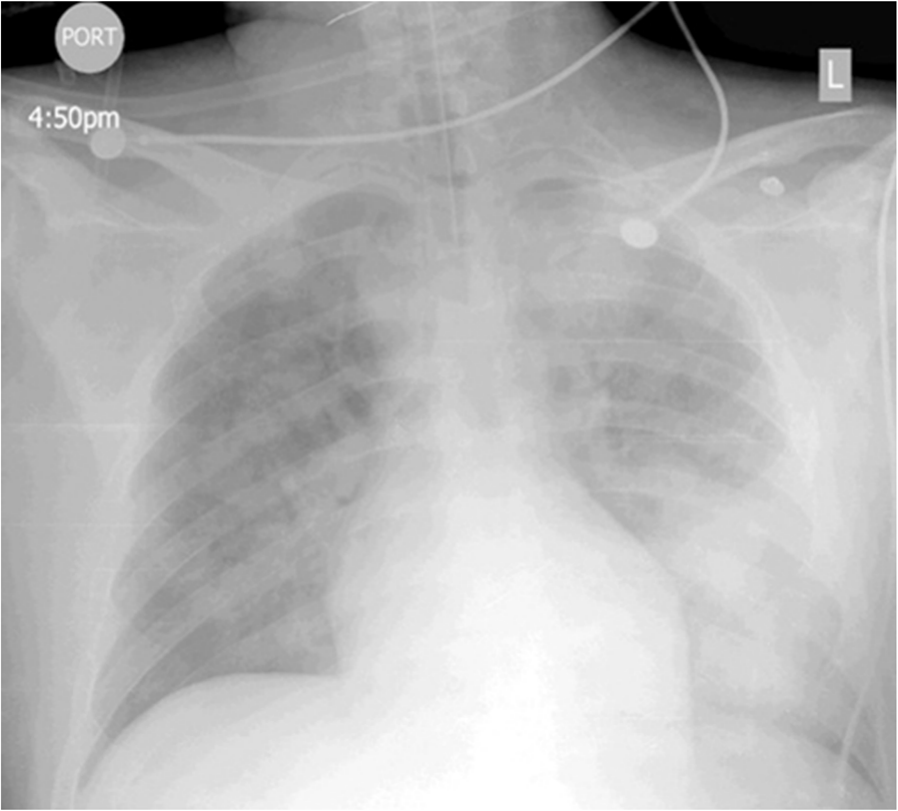
Post-intubation chest X-ray demonstrating diffuse bilateral alveolar infiltrates left more than right.

A 24-year-old Hispanic man presented after a witnessed tonic–clonic seizure with urinary incontinence that resolved after lorazepam administration. He had a remote history of seizures but was not on any medications. He was postictal, afebrile and tachycardic with a pulse of 124 beats per minute (bpm) and blood pressure (BP) of 144/66mmHg. Initial laboratory was remarkable for a lactate of 17mmoles/L, which resolved rapidly. A routine urine toxicology screen was positive for cannabinoids and benzodiazepines and negative for cocaine, phencyclidine, opiates and amphetamines. The next day he complained of abdominal pain, diarrhea, vomiting and developed progressive agitation and confusion. His lipase level was normal; however, his serum creatinine had increased from 1.4 to 4.3g/dL and subsequently increased further (Table 1). Within a few hours his respiratory distress worsened, becoming hypoxic with an arterial saturation of 70%. Also noted were a fever of 38.89°C (102°F), heart rate of 140bpm, and BP of 163/104mmHg. After failing a trial of non-invasive ventilation, he was intubated. A chest X-ray revealed bilateral infiltrates (Figure 1). He remained hypoxemic after intubation requiring 100% fraction of inspired oxygen. Despite not meeting compliance criteria for acute respiratory distress syndrome his oxygenation responded to high positive end-expiratory pressure. Antibiotics were initiated for possible pneumonia. Bronchoscopy revealed normal airways and clear secretions, not compatible with aspiration or bacterial pneumonia. Bronchoalveolar lavage was performed. Subsequently, an echocardiogram demonstrated an ejection fraction of 30% with wall motion abnormalities. Bronchoscopy and echocardiogram findings suggested the etiology of hypoxemic respiratory failure was most likely fluid overload. The etiology for acute kidney injury (AKI) and congestive heart failure (CHF) was unclear. After three sessions of hemodialysis, his oxygenation, urine output, and creatinine levels improved. He was extubated on hospital day eight allowing further history. Apparently several hours prior to symptom onset he had consumed the street drug “Molly” with alcohol, marijuana, and “purple drank”, a slang term for a drink containing codeine and promethazine. No further hemodialysis was required and a repeat echocardiogram demonstrated normalization of his left ventricular function. He was discharged home on day twelve of hospitalization.

**Table 1:** Pertinent laboratory values by hospital days

**Day of hospitalization**	**Day 0**	**Day 3**	**Day 12**
pH	7.09	7.42	7.45
Carbon dioxide partial pressure	38.9	26.7	33.9
Oxygen partial pressure	112	43.8	78.7
Serum bicarbonate (mEq/L)	11	21	23
Lactic acid (mmoles/L)	17	3.6	0.9
Blood urea nitrogen (g/dL)	12	43	35
Serum creatinine (g/dL)	1.4	8.2	1.7
Serum sodium (mEq/L)	137	129	141
Potassium (mEq/L)	4.0	4.0	3.7
Creatine kinase (units/L)	473	863	223
Hemoglobin (g/dL)	15	13.5	12.8
White blood cell (k/μL)	17.8	12.2	14.1
Platelets (k/μL)	420	259	328

## Discussion

In summary, our patient was a young man presenting with a seizure and subsequent multiorgan failure. Several hypotheses were considered to explain his presentation including aspiration pneumonitis, severe sepsis, neuroleptic malignant syndrome, rhabdomyolysis leading to AKI and collagen vascular disease with diffuse alveolar hemorrhage. There was no clear source of infection and all cultures including bronchoalveolar lavage were negative. Although he did receive one dose of haloperidol intravenously for his agitation on day 1, neuroleptic malignant syndrome was unlikely as he was only briefly febrile, there was no rigidity and creatine kinase levels were only mildly elevated. Collagen vascular disease seemed unlikely, as all his serologic markers were negative. Of interest, he had a complete and rapid recovery with just supportive treatment. This presentation could be explained by drug toxicity. The initial drug screen was negative for amphetamines. However, this was performed by routine immunoassay, which has a low sensitivity for detecting MDMA. Severity of patient presentation is not necessarily related to the ingested dose of MDMA [3,4]. Our patient admitted taking multiple drugs along with “Molly”. However, it is unlikely that the other substances ingested concomitantly with “Molly” in our patient would have contributed to a rapid onset multisystem organ failure.

MDMA toxicity is due to release of neurotransmitters such as serotonin and dopamine causing hyperthermia, seizures and muscle breakdown leading to renal failure [1,2]. Cardiac toxicity can lead to CHF, arrhythmias and myocardial infarction. The mechanism of cardiac toxicity is not clearly understood but can be related to oxidative stress from toxic metabolites of MDMA [5,6]. In addition, other toxic effects include hyponatremia, disseminated intravascular coagulation, liver failure and cerebrovascular accidents [7,8]. The development of hyponatremia is multifactorial and is thought to be primarily due to drug-induced excessive thirst and secretion of arginine vasopressin in the setting of fluid availability [6]. Various constellations of these manifestations have resulted in case reports of multisystem organ failure.

Several mechanisms of AKI associated with MDMA have been proposed with the most common etiology being seizure-precipitated rhabdomyolysis potentially accentuated by volume depletion and hypotension. These cases have pronounced levels of creatine kinase [6]. In our patient the cause of renal failure was not clear given only mildly elevated creatine kinase levels. As shown in Table 1, a creatine kinase level of 863 units/L was the highest level recorded during his entire hospitalization. Retrospectively, he most probably developed drug-induced renal artery vasoconstriction leading to renal hypoperfusion and reversible acute tubular necrosis. Severe symptomatic hyponatremia is another serious complication of MDMA that can present with mental status changes and seizures. Although our patient presented with a seizure, it did not appear to be related to hyponatremia nor to hyperthermia. His confusion and agitation prior to intubation could be interpreted as drug withdrawal but was more probably attributable to encephalopathy in the setting of AKI and hypoxemia. Acute respiratory failure in the context of multisystem failure is due to acute lung injury in most cases discussed in the literature [9]. In our patient this appeared more related to fluid overload as a consequence of CHF and AKI.

Based on a review of case reports, Liechti *et al*. described two common presentations of ecstasy-related death: the first being multiorgan failure precipitated by hyperthermia and the second, hyponatremia-induced brain edema [10]. Our patient had only a transient fever spike with minimally decreased sodium; he does not appear to fit into either of these categories.

Contrary to the belief that “Molly” is pure MDMA, it is often adulterated with so-called designer drugs, for example methylenedioxypyrovalerone also known as bath salts. These compounds inhibit norepinephrine-dopamine reuptake and have a side-effect profile similar to MDMA including AKI [11]. It is possible that our patient unknowingly ingested bath salts with “Molly”.

## Conclusions

Physicians evaluating patients with increased sympathetic activity and multisystem failure should consider drug intoxication in the differential especially when other etiologies become less likely. Rapid and complete reversal of multisystem failure with supportive treatment only is suggestive of drug intoxication. Often, as in our case, a full history has to be elicited retrospectively. Therefore when drug intoxication is considered a full toxicology screen including MDMA levels should be obtained. Routine immunoassays for detecting amphetamines are unreliable in detecting MDMA and more specific screening tests should be requested. In patients with “Molly”-induced organ failure it is difficult to predict which organs may fail [12]. Pathophysiology and severity of organ failure may vary.

“Molly” is abused in our community. Sadly, young people who consume a single dose are at risk of serious side effects because, unlike other drugs, the toxicity of MDMA is not necessarily dose related [12]. In addition, unknowing consumption of harmful adulterants may also occur. “Molly” may be used in conjunction with other recreational drugs with their own unique toxicities [13]. Increased awareness of the dangers of “Molly” is needed among the youth of our community.

## Consent

Written and informed consent was obtained from the patient for publication of this case report and accompanying image. A copy of the written consent is available for review by the Editor-in-Chief of the Journal.

## Abbreviations

AKI: Acute kidney injury; BP: Blood pressure; bpm: Beats per minute; CHF: Congestive heart failure; MDMA: 3,4-methylenedioxymethamphetamine.

## Competing interests

The authors declare that they have no competing interests.

## Authors’ contributions

TV and MD wrote the initial draft of the manuscript. RD and KU revised the manuscript critically for important intellectual content and gave final approval of the version to be published. All authors read and approved the final manuscript.
